# Integrative analysis of microRNA-320a-related genes in osteoarthritis cartilage

**DOI:** 10.3389/fsurg.2022.1005243

**Published:** 2023-01-09

**Authors:** Hao Peng, Haibin Lin

**Affiliations:** ^1^ The Third Clinical College of Southern Medical University, Guangzhou, China; ^2^ Department of Orthopedics, Affiliated Hospital of Putian University, Putian, China

**Keywords:** osteoarthritis, bioinformatics, microRNA-320a, go, KEGG, PPI

## Abstract

**Objectives:**

To investigate microRNA-320a-related differentially expressed genes (DEGs) and pathways in osteoarthritis (OA) by bioinformatic analysis.

**Methods:**

The target genes of microRNA-320a were searched and collected from MiRTarBase microRNA Targets dataset, the TargetScan Predicted Nonconserved microRNA Targets dataset and the TargetScan Predicted Conserved microRNA Targets dataset. OA-related microRNAs and OA-related target genes were collected from GeneCards databases. The pathway enrichment analysis of miRNAs ware performed by Funrich analysis tool. Gene Ontology (GO) and Kyoto Encyclopedia of Genes and Genomes (KEGG) analysis was obtained from Database for Annotation, Visualization and Integrated Discovery (DAVID). GeneMANIA and STRING are used for protein-protein interaction (PPI) network analysis. Module analysis was performed by Cytoscape.

**Results:**

A total of 176 OA related miRNAs were searched and collected for enrichment analysis, and microRNA-320a was one of OA related miRNAs. Enrichment pathway and analysis of 1721 miRNA-320a-related target genes from MiRTarBase and TargetScan were performed using the online tools Metascape. And results shown that the biological processes were remarkably enriched in chromatin organization, cellular response to DNA damage stimuli, mRNA metabolic process, protein ubiquitination, and regulation of cell adhesion. And then we analysed miRNA-320a-targeted OA genes via KEGG, GO enrichment and PPI Network. Our results showed that miRNA-320a played a role in OA through FoxO signaling pathway, PI3K-Akt signaling pathway, focal adhesion, MAPK signaling pathway, HIF-1 signaling pathway and cellular senescence. And we speculate that MAPK signaling pathway plays a key role in the effect of miRNA-320a on OA.

**Conclusion:**

This study implied microRNA-320a-related DEGs and dysregulated pathways in OA. The aim is to screen miRNA-320a-related genes and pathways in OA and, eventually, to improve the understanding of underlying mechanisms of miRNA-320a in OA.

## Introduction

As one of the most common chronic degenerative articular cartilage diseases, osteoarthritis (OA) has a positive correlation with age, and its incidence is increasing, seriously affecting people's quality of life and physical and mental health, and causing a heavy burden to families, society and the country. The main pathological features of OA include wear and tear of articular cartilage, degeneration and excessive apoptosis of chondrocytes, degeneration of articular chondrocytes (1–3).

MiRNA (MicroRNA) is a class of endogenous small RNAs with about 20–24 nucleotides in length, and plays important roles in cell differentiation, biological development and disease occurrence and development. Bastami et al. developed a bioinformatics method to identify miRNAs target sites polymorphisms related to obesity or colorectal cancer (CRC), which could provide potential biomarkers for diagnoses, prognoses and treatment for CRC or obesity (4). And exosomal miRNAs are associated with many diseases, and provided diagnoses potential biomarkers and therapy targets for diseases (5). The pathogenesis of osteoarthritis is a very complex process involving many aspects, including cartilage degradation and gene expression changes in chondrocytes, including miRNA changes, with familial heritability (6). More and more studies have shown that miRNA plays an important regulatory role in the pathological development of OA. For example, miR-495 inhibits chondrogenic differentiation, and miR-127 regulates the MMP-13 expression (7, 8). miRNA-320a is closely related to the pathogenesis of a variety of diseases, including tumors, immune diseases, inflammatory diseases and so on (9, 10). Wang et al. reported that miRNA-320a inhibited cell growth ability, migration, and invasion by targeting the 3′-UTR of cytoplasmic polyadenylation element-binding protein 1 (CPEB1) in osteosarcoma, which might as a potential therapy target for osteosarcoma (11). And miRNA-320a has also been shown to regulate the expression of MMP-13 in chondrogenesis and interleukin-1β (IL-1β)-induced chondrocyte responses. Therefore, the specific mechanism of miRNA-320a in the pathogenesis of osteoarthritis is the focus of current research.

In this study, MiRTarBase microRNA Targets dataset, TargetScan Predicted Nonconserved microRNA Targets dataset and the TargetScan Predicted Conserved microRNA Targets dataset were analyzed to identify the target genes of miRNA-320a. Subsequently, GO and KEGG pathways were enriched, PPI network was established, and modules were analyzed. OA related miRNAs and target genes were collected from GeneCards. Enrichment pathway and analysis were performed using the online tools Metascape. This work aims to screen miRNA-320a-related genes and pathways in OA and, eventually, to improve the understanding of underlying mechanisms of miRNA-320a in OA.

## Methods

### Database establishment

The target genes of the microRNA-320a were searched and collected from MiRTarBase microRNA Targets dataset (http://mirtarbase.mbc.nctu.edu.tw/php/index.php), the TargetScan Predicted Nonconserved microRNA Targets dataset and the TargetScan Predicted Conserved microRNA Targets dataset (https://www.targetscan.org/vert_80/). OA-related microRNAs and OA-related target genes were collected from GeneCards databases (https://www.genecards.org/).

### Functional enrichment analysis

The pathway enrichment analysis of miRNAs ware performed by Funrich analysis tool. The difference was statistically significant at *P* < 0.05. Enrichment Pathway and Analysis were performed by Metascape (https://metascape.org/gp/index.html#/main/step1). Gene Ontology (GO) and Kyoto Encyclopedia of Genes and Genomes (KEGG) analysis was obtained from DAVID (https://david.ncifcrf.gov/), and the visualization of the signaling pathways was drawn by bioinformatics (http://www.bioinformatics.com.cn/).

### Gene ontology (GO) and Kyoto encyclopedia of genes and genomes (KEGG) enrichment analyses

In order to predict protein-protein interaction (PPI), GeneMANIA in Cytoscape is used for PPI network analysis. Nodes represent genes and edges represent connections between genes. Molecular complex detection (MCODE) plug-in component in Cytoscape v3.8.2 software is used to find key sub-networks and genes in a huge network according to the relationship between edges and nodes (degree cutoff = 2, node score cutoff = 0.2, *k*-core = 2, and max.depth = 100), so as to facilitate downstream analysis.

## Results

### Enrichment analysis of OA-related miRNAs

**Figure 1:**
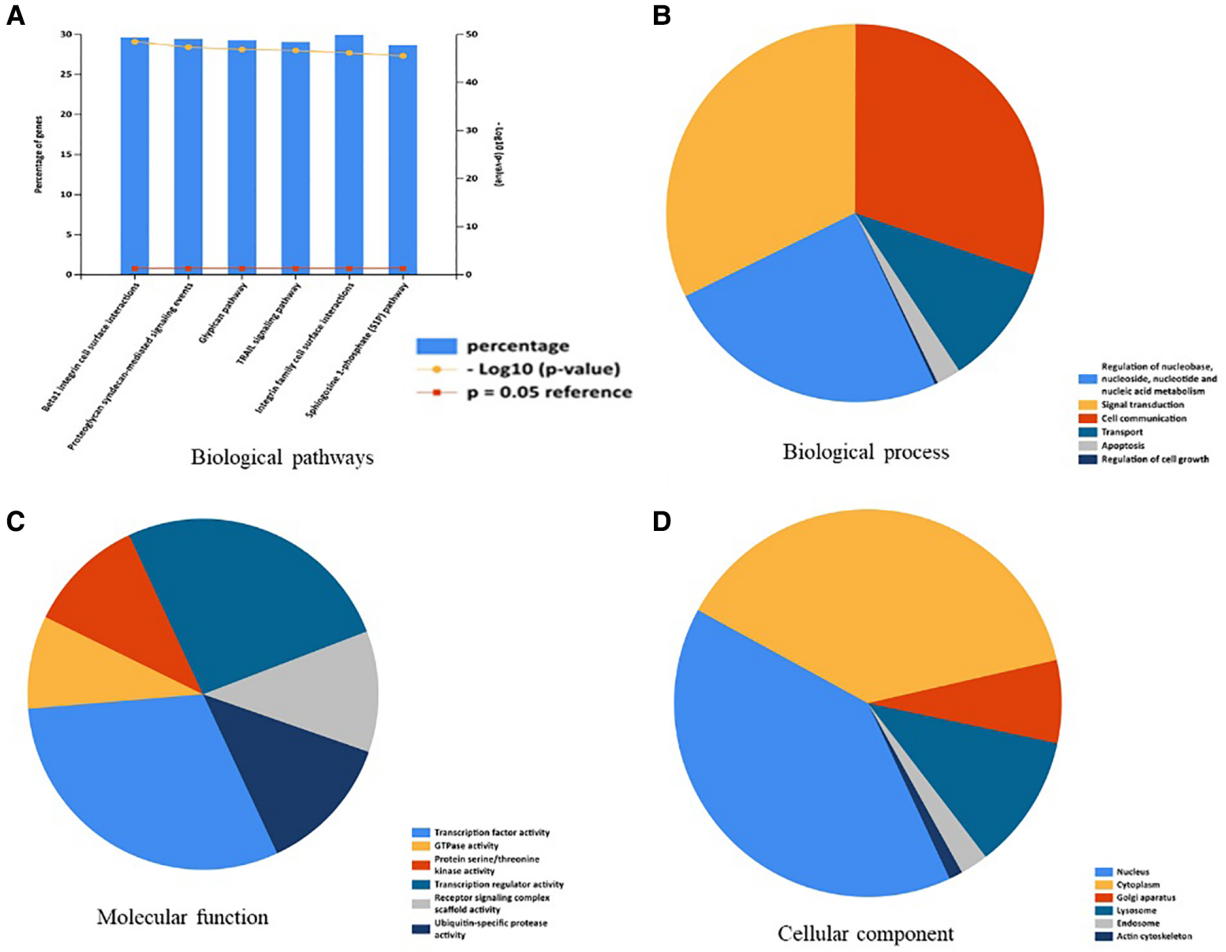
Enrichment analysis of OA-related miRNAs. (**A**) Biological pathways were analysis by Funrich. (**B**) Biological process was analysis by Funrich. (**C**) Molecular function was analysis by Funrich. (**D**) Cellular component was analysis by Funrich.

A total of 176 OA related miRNAs were searched and collected from the GeneCards, and were all uploaded to Funrich. The results of enrichment analysis on biological pathway were mainly enriched in Btra1 integrin cell surface interactions, proteoglycan syndecan-mediated signaling events, glypican pathway, TRAIL signaling pathway, integrin family cell surface interactions, and sphingosine 1-phosphate (S1P) pathway (Figure 1A). The biological process enriched included regulation of nucleobase, nucleoside, and nucleic acid metabolism, signal transduction, cell communication, transport, apoptosis, and regulation of cell growth (Figure 1B). The molecular function enriched included transcription factor activity, GTPase activity, protein serine/threonine kinase activity, transcription regulator activity, receptor signaling complex scaffold activity, and ubiquitin-specific protease activity (Figure 1C). The cellular component enriched mainly included nucleus, cytoplasm, golgi apparatus, and lysosome (Figure 1D).

### Enrichment pathway and analysis of miRNA-320a-targeted genes

**Figure 2:**
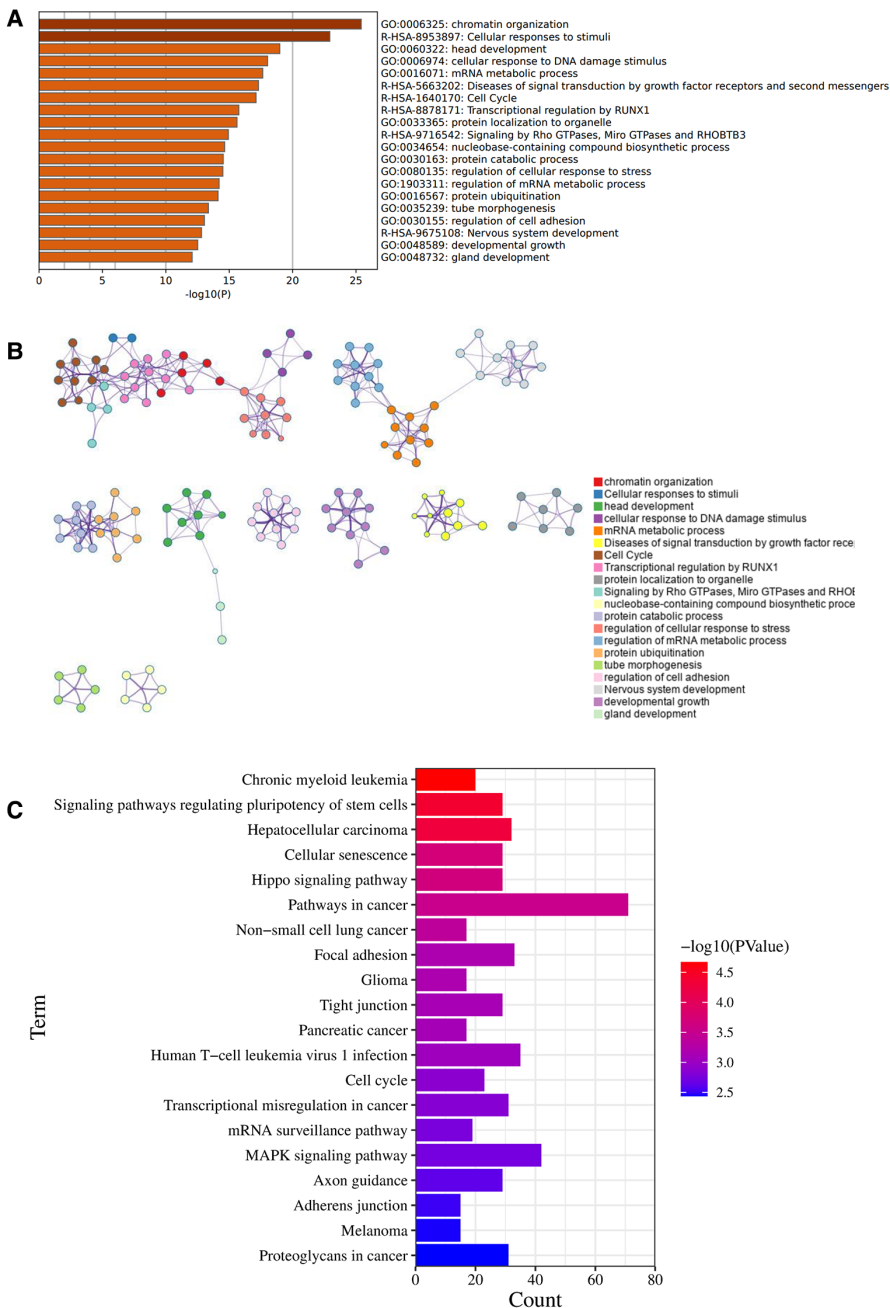
Enrichment pathway and analysis of microRNA-320a-targeted genes. (**A**) Bar graph of 20 enriched terms were colored by *p*-values, and *p*-value < 0.01 was statistically significant. (**B**) Network of enriched terms colored by cluster ID, where nodes sharing the same cluster ID are usually close to each other. (**C**) The top 20 remarkably enriched signaling pathway were analysis by KEGG analysis.

Between these OA related miRNAs, we focus on the role and mechanism of miRNA-320a in OA. We searched and collected target genes of miRNA-320a from MiRTarBase and TargetScan. 480 target genes of the miRNA-320a were obtained from the MiRTarBase microRNA Targets dataset, 788 target genes of the miRNA-320a predicted using conserved miRNA seed sequences and 535 target genes of the miRNA-320a predicted using nonconserved miRNA seed sequences were obtained from the TargetScan microRNA Targets dataset. By integrating these data, we get 1,721 target genes of the miRNA-320a. And then Pathway enrichment analyses of miRNA-320a-targeted genes were performed using the online tools Metascape. It was shown that the biological processes were remarkably enriched in chromatin organization, cellular response to DNA damage stimuli, mRNA metabolic process, protein ubiquitination, and regulation of cell adhesion. The biological pathway was significantly enriched in cellular responses to stimuli, cell cycle, transcriptional regulation by RUNX1, signaling by Rho GTPases, Miro GTPases and RHOBTB3 (Figures 2A,B). KEGG functional analysis revealed that the miRNA-320a-targeted genes were significantly enriched in signaling pathways regulating pluripotency of stem cells, cellular senescence, hippo signaling pathway, focal adhesion, tight junction, cell cycle, mRNA surveillance pathway, MAPK signaling pathway and adherens junction (Figure 2C).

### Enrichment pathway and analysis of OA-related genes

**Figure 3:**
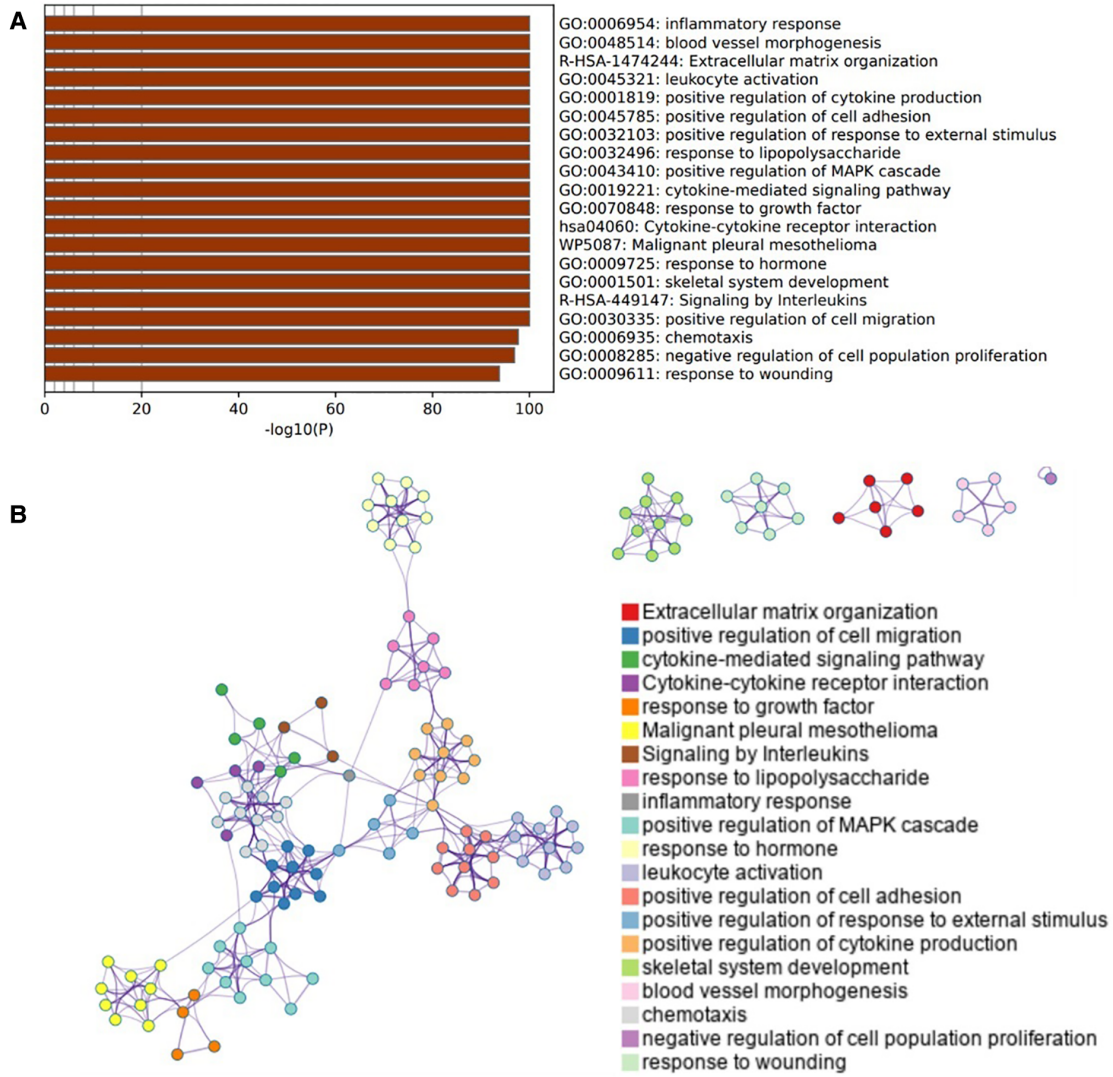
Enrichment pathway and analysis of OA-related genes. (**A**) Bar graph of 20 enriched terms were colored by *p*-values, and *p*-value < 0.01 was statistically significant. (**B**) Network of enriched terms colored by cluster ID, where nodes sharing the same cluster ID are usually close to each other.

3,183 OA-related target genes were collected from GeneCards. And then Pathway enrichment analyses of OA-related target genes were performed using the online tools Metascape. It was shown that the biological processes were remarkably enriched in inflammatory response, positive regulation of cytokine production, cell adhesion, Mitogen-activated protein kinases (MAPK) cascade and cell population proliferation. The biological pathway was significantly activated in extracellular matrix organization, cytokine-cytokine receptor interaction, and signaling by interleukins (Figures 3A,B).

### KEGG and go pathway enrichment analysis of miRNA-320a-targeted genes within OA-related genes (miRNA-320a-targeted ORG)

**Figure 4:**
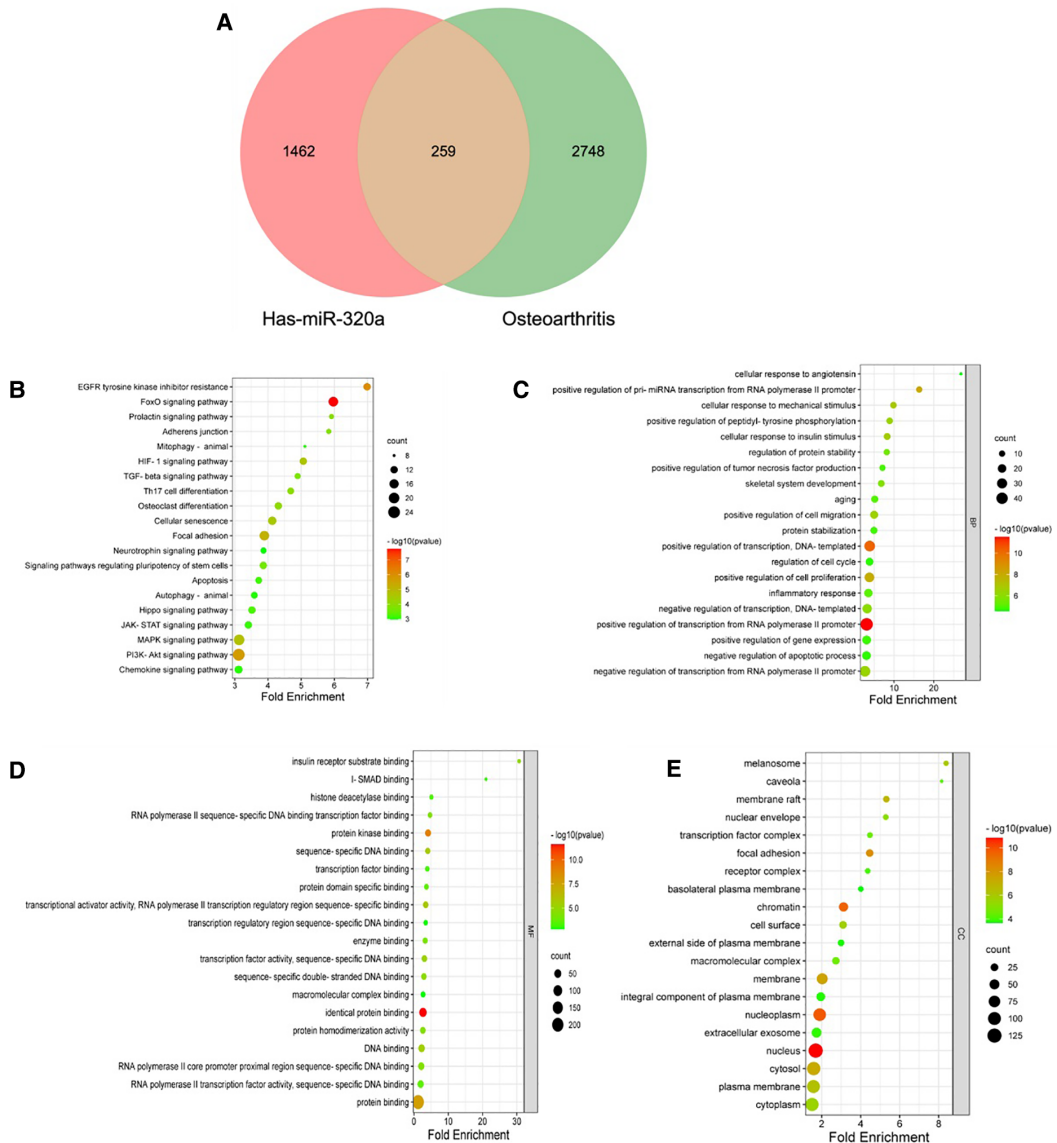
KEGG and GO pathway enrichment analysis of microRNA-320a-targeted genes which have the effect on OA. (**A**) PPI network was obtained from GeneMANIA database. The nodes represent genes and the edges represent connection between genes. Five key modules were identified by MCODE, which was used for network gene clustering. (**B**) Cluster 1 with 11.067 score. (**C**) Cluster 2 with 9.579 score. (**D**) Cluster 3 with 6.1 score. (**E**) Cluster 4 with 3.778 score. (**F**) Cluster 5 with 2.5 score. (**G**) Pathway enrichment analysis of cluster 1. The MAPK signaling pathway was significantly enriched. (**H**) Pathway enrichment analysis of cluster 2. The MAPK signaling pathway was significantly enriched.

We intersected miRNA-320a-targeted genes with OA-related genes. Based on overlapping results in the Venn diagrams (Figure 4A), 259 genes were identified. We first used David online database for miRNA-320a-targeted ORG pathway enrichment analysis. As shown in Figure 4B, miRNA-320a played a role in OA through FoxO signaling pathway, PI3K-Akt signaling pathway, Focal adhesion, MAPK signaling pathway, HIF-1 signaling pathway and Cellular senescence. And then we conducted GO enrichment analysis from three functional categories, including BP, MF and CC. The biological process of these genes mainly included positive regulation of transcription from RNA polymerase II promoter, transcription, pre-miRNA transcription from RNA polymerase II promoter and cell proliferation, inflammatory response, and age (Figure 4C). The molecular functions of these genes mainly included identical protein binding, protein kinase binding, transcriptional activator activity, DNA binding, and transcription factor activity (Figure 4D). The cellular component of these genes mainly included nucleus, nucleoplasm, chromatin, focal adhesion, membrane, and cytosol (Figure 4E).

### Protein–protein interaction (PPI) network analysis of miRNA-320a-targeted ORG

**Figure 5:**
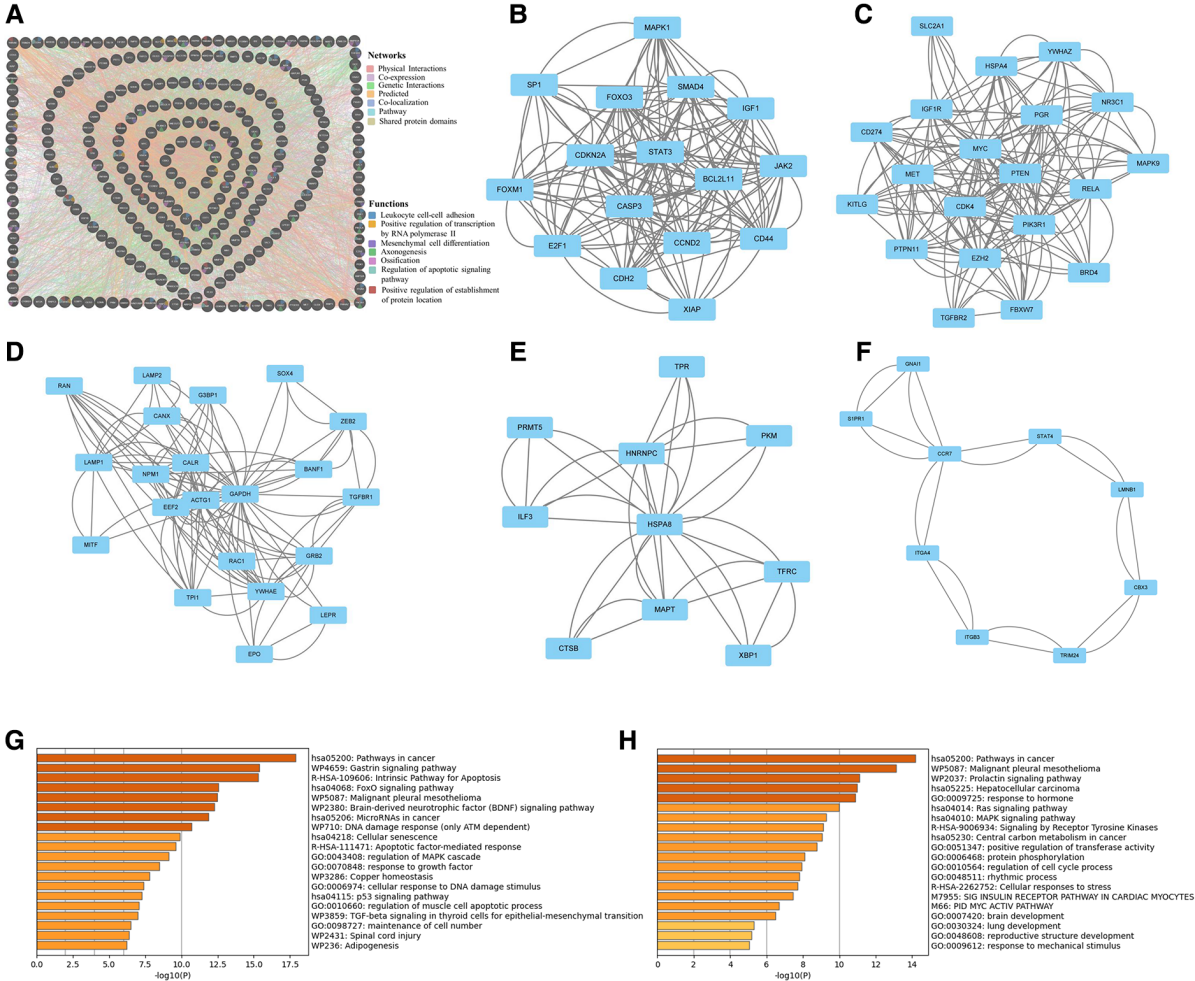
Protein–protein interaction network analysis of microRNA-320a-targeted genes which have the effect on OA. (**A**) PPI network was obtained from GeneMANIA database. The nodes represent genes and the edges represent connection between genes. Five key modules were identified by MCODE, which was used for network gene clustering. (**B**) Cluster 1 with 11.067 score. (**C**) Cluster 2 with 9.579 score. (**D**) Cluster 3 with 6.1 score. (**E**) Cluster 4 with 3.778 score. (**F**) Cluster 5 with 2.5 score. (**G**) Pathway enrichment analysis of cluster 1. The MAPK signaling pathway was significantly enriched. (**H**) Pathway enrichment analysis of cluster 2. The MAPK signaling pathway was significantly enriched.

We obtained the PPI network from by GeneMANIA. Our study showed that physical interactions account for 56.93%, co-expression accounts for 25.69%, genetic interactions account for 7.76%, predicted accounts for 4.18%, co-localization accounts for 3.78%, pathway accounts for 1.63, and shared protein domains account for 0.04% (Figure 5A). To further examine the key modules, we used the application of MCODE from Cytoscape to facilitate clustering analysis of gene networks. Five key modules with 76 genes were established, and the scores were 11.067, 9.579, 6.1, 3.778 and 2.5 respectively (Figures 5B–F). Pathway enrichment analyses of top two key modules were performed using the Metascape. It showed that the pathway of genes in modules-1 were mainly involved intrinsic pathway of apoptosis, FoxO signaling pathway, cell senescence, and regulation of MAPK cascade (Figure 5G). And the pathway of genes in modules-2 were mainly involved Ras signaling pathway, MAPK signaling pathway, signaling by receptor tyrosine kinases, regulation of cell cycle process, and response to mechanical stimulus (Figure 5H). Form these results, we speculate that MAPK signaling pathway plays a key role in the effect of miRNA-320a on OA.

## Discussion

OA is the most common joint disease in the elderly population. Affected by age, gender, obesity, genetics, mechanical stress and other pathogenic factors, the incidence of OA is getting higher and higher. With the increase of age, the disease gradually worsens, resulting in joint deformity and seriously affecting the quality of life of patients (12, 13). At present, the understanding of the pathogenesis of OA is still incomplete, and the treatment options provided to patients still have certain limitations, which often cannot effectively curb the development of OA (14).

A total of 176 OA related miRNAs were searched and collected from the GeneCards. Biological pathways, biological processes and cell components were enriched and analyzed. Pathway analysis demonstrated that Btra1 integrin cell surface interactions, proteoglycan syndecan-mediated signaling events, glypican pathway, TRAIL signaling pathway, integrin family cell surface interactions, and sphingosine 1-phosphate (S1P) pathway play important roles. OA is associated with extracellular matrix (ECM) changes and tissue degeneration. The integrin protein family connects structural information in the extracellular matrix of OA to the complex response mechanisms inside the cell (15). Fibronectin (Fn) is a glycoprotein that is highly expressed in arthritic joints and mediates various physiological processes by regulating interactions between cell surface integrin receptors and growth factors (16). Because a single miRNA can regulate a large number of genes, and a single molecule can be targeted by many miRNAs, it is a rather complex cross-chain regulatory network (17).Therefore, analyzing the overall expression profile of miRNAs during OA may help us better understand the pathogenesis of OA.

1,721 target genes of miRNA-320a from MiRTarBase and TargetScan were performed using the online tools Metascape. It was shown that the biological processes were remarkably enriched in chromatin organization, cellular response to DNA damage stimuli, mRNA metabolic process, protein ubiquitination, and regulation of cell adhesion. The biological pathway was significantly enriched in cellular responses to stimuli, cell cycle, transcriptional regulation by RUNX1, signaling by Rho GTPases, Miro GTPases and RHOBTB3. MiRNA silences gene expression through transcription, post-transcription, and chromatin dependent pathways (18, 19). Aging is associated with the pathophysiology of OA, and miRNAs are closely associated with the control of aging (20, 21). Studies have found that the growth factors released from the degraded matrix during early OA provide a powerful pro-mitotic stimulus, and the mitotic stimulation of damaged chondrocytes induces senescence (22–24). Runx1 has been shown to be an important factor in early chondrogenic differentiation and can restore cartilage anabolism by promoting chondrocyte differentiation (25, 26). The target genes of miRNA-320a are closely related to OA, suggesting that miRNA-320a might play important roles in the occurrence and development of osteoarthritis.

Many signaling pathways are involved in the progression of OA (27–33), including NF-*κ*B signaling pathway, JNK signaling pathway, MAPK signaling pathway, Wnt signaling pathway, PI3K/AKT/mTOR signaling pathway, and so on. Among them, NF-*κ*B signaling pathway is reported to be involved in chondrocyte catabolism, chondrocyte survival and synovial inflammation (30). And MAPK signaling pathway is reported to be play a pivotal role in inflammatory factor production and the cartilage degeneration process in OA (28). Researches on signaling pathways involved in regulating the progression of OA will provide potential therapeutic targets for OA. Our current study showed that there are 259 miRNA-320a-targeted genes in OA related genes. As shown in Figure 4B, miRNA-320a played a role in OA through FoxO signaling pathway, PI3K-Akt signaling pathway, Focal adhesion, MAPK signaling pathway, HIF-1 signaling pathway and Cellular senescence. FoxO is an evolutionarily conserved transcription factor family, which consists of four members (FoxO1, FoxO3, FoxO4 and FoxO6) (34, 35). FoxO transcription factor can prevent aging of cells and organisms, and its expression in cartilage is reduced with aging and in OA (36). A large number of miRNAs can directly regulate FOXO transcripts (37). PI3K/AKT/mTOR is an important and very complex signaling pathway, and more and more evidences support the involvement of PI3K/AKT/mTOR in the development of OA (38, 39). Compared with normal cartilage, the PI3K/AKT pathway was down-regulated in OA cartilage tissue and involved in ECM synthesis (40). Evidences have shown that microRNAs play important roles in regulating PI3K/AKT/mTOR pathway and its impact on OA development, suggesting that microRNAs may become potential therapeutic targets for OA (41, 42).

PPI Network Analysis of miRNA-320a-targeted ORG also showed that MAPK signaling pathway plays a key role in the effect of miRNA-320a on OA. MAPK serine/threonine protein kinase family is widely present in eukaryotes and regulates a variety of cellular processes including proliferation, differentiation, survival and apoptosis (43, 44). LAN et al. showed that MAPKs inhibition had a protective effect on OA by restoring damaged OA autophagy (45). MiRNA-320a is regulated by the P38 MAPK/JNK pathway in tumor cells (46). Inhibition of miRNA-320a synthesis through AMPK and P38 signaling pathway may improve the pathogenesis of OA (47).

MiRNA-320a is a very important miRNA that can regulate the expression of multiple target genes. This study implied microRNA-320a-related DEGs and dysregulated pathways in OA. The aim is to screen miRNA-320a-related genes and pathways in OA and, eventually, to improve the understanding of underlying mechanisms of miRNA-320a in OA.

## Data Availability

The original contributions presented in the study are included in the article/Supplementary Material, further inquiries can be directed to the corresponding author.
